# Shared System-Level Drivers of Unprofessional Conduct During Clinical Rotations Among Medical and Dental Students: An Interpretive Description Study

**DOI:** 10.7759/cureus.101650

**Published:** 2026-01-15

**Authors:** Hassan Jan, Malik Zain Ul Abideen, Maira Sahar Malik, Laila Ajmal, Usman Ul Haq, Qurat Ul Ain Mehfooz, Jawad Tareen

**Affiliations:** 1 Department of Operative Dentistry, Peshawar Dental College, Peshawar, PAK; 2 Department of Dental Education and Research, Bakhtawar Amin Medical and Dental College, Multan, PAK; 3 Department of General Medicine, Foundation University Medical College, Islamabad, PAK; 4 Department of Paedodontics, CIMS Dental College, Multan, PAK; 5 Department of Oral and Maxillofacial Surgery, Wah Medical College, Wah Cantt, PAK; 6 Department of Medical Education, Bakhtawar Amin Medical and Dental College, Multan, PAK

**Keywords:** clinical rotations, dental students, health professions education, hidden curriculum, medical students, professionalism, qualitative research, unprofessional behaviour

## Abstract

Introduction

Unprofessional conduct in clinical education refers to behaviours that deviate from accepted professional norms. These behaviours undermine learner well-being, patient dignity, and the development of professional identity. Findings from previous literature suggest that unprofessional conduct is common in Pakistani clinical training and may be reinforced within clinical environments, potentially through the influence of negative elements of the hidden curriculum. While individual incidents are often reported, less is known about how institutional and system-level factors interact to normalise or hinder mistreatment in low-resource clinical education settings. Therefore, this study aimed to explore final-year medical and dental students' perceptions of shared, system-level drivers of unprofessional conduct during clinical rotations, with the goal of producing practice-oriented recommendations for clinical education.

Methodology

This interpretive description study was conducted at a private medical and dental college in Multan, Pakistan. Participants were recruited with purposive sampling that included final-year Bachelor of Medicine, Bachelor of Surgery (MBBS) and Bachelor of Dental Surgery (BDS) students who had completed clinical rotations and consented to audio-recording. Data collection continued until interpretive sufficiency was reached. Semi-structured, in-depth interviews were conducted face-to-face using a pilot-tested, expert-validated guide. Recordings were transcribed manually, and data were analysed using iterative immersion, open coding, constant comparison, development of analytic categories, and construction of higher-order themes consistent with interpretive description.

Results

Interviews revealed five interrelated thematic patterns: (1) hierarchy and power, including unchecked senior authority, social bias, and unfair treatment with paramedical staff; (2) hidden curriculum and norms, including silence and obedience, compensatory acts by clinicians, haphazard training, and informal practices that contradicted formal professionalism teaching; (3) systemic pressures and drivers, including excessive patient loads, resource scarcity and dual-practice incentives which shifted faculty priorities away from clinical teaching and promoted expedient, and instrumental behaviours; (4) challenges to patient-centred practice, where patients were often objectified during teaching, with inadequate communication, privacy breaches, and gendered biases; and (5) student impact and coping, where exposure to unprofessional conduct produced shame, moral distress, and withdrawal, while some students expressed a strong desire for institutional change. Together, these themes indicate that unprofessional behaviour is systemically embedded and socially learned rather than solely individual failings.

Conclusions

Unprofessional conduct during clinical rotations reflects the combined influence of unregulated power, negative role-modelling in hidden curricula, and system-level constraints rather than isolated individual failings. Addressing these issues requires institutional strategies that protect teaching time, promote respectful supervision, and provide safe, confidential reporting mechanisms. Such approaches can strengthen learner well-being, uphold patient dignity, and reinforce professionalism in clinical education.

## Introduction

Professionalism refers to the consistent demonstration of competence, ethical conduct, accountability, and responsibility in roles that carry public trust and specialised expertise [1]. In the health professions, professionalism is a multifaceted construct encompassing the values, attitudes, and behaviours required for safe and ethical healthcare delivery [2]. These include altruism, respect, integrity, responsibility, and compassion, which complement clinical expertise [2]. The health professions learning environment, composed of physical, social, and psychological elements, plays a central role in shaping how students develop both academically and professionally [3]. Healthcare professionals are among the most trusted members of society, and patient trust is closely linked to perceptions of professional behaviour [4]. Accordingly, professionalism is widely recognised as a core competency in medical and dental education and is explicitly included among fundamental domains by accreditation bodies such as the Accreditation Council for Graduate Medical Education (ACGME) [4]. Developing professionalism in medical and dental students is therefore essential for ethical practice and for sustaining social responsibility of the profession [4,5].

The clinical workplace exposes students to everyday norms and practices of healthcare through its teams, routines, and organisational culture [6]. Through ongoing interactions with peers, senior clinicians, and patients, students are socialised into professional roles as part of a community of practice [7]. Alongside formal teaching, an informal or hidden curriculum operates, consisting of unwritten lessons conveyed through institutional policies, role-modelling, and daily clinical routines [8]. In clinical training settings, negative elements of the hidden curriculum can undermine formal professionalism teaching and contribute to the erosion of professional values [8].

Together, the explicit and hidden curricula shape learners' professional identity. Previous research indicates that students frequently identify the learning environment, especially the hidden curriculum and observed staff behaviours, as a major influence on their professional identity formation [9]. A study reported that students highlighted the strong influence of staff behaviours, transmitted through the hidden curriculum, on their attitudes and practice [10].

Unprofessional conduct, defined as behaviours observed in clinical practice that contravene accepted professional norms, is frequently reported in the literature [11]. A range of unprofessional behaviours among healthcare staff and trainees has been documented across clinical settings [11]. A systematic review of student lapses grouped numerous incidents into categories such as "failure to engage" (e.g., absenteeism and inattention), "dishonesty" (e.g., data fabrication), "disrespect" (e.g., rude communication), and "poor self-awareness" [12]. Recent surveys of early-career clinicians report high awareness of professionalism alongside frequent observation of unprofessional acts, including neglect of duties and informal patient practices [13]. Locally, a large cross-sectional study of medical undergraduates in Pakistan found that approximately 70% of students scored within a range interpreted as indicating an "unprofessional attitude", with higher prevalence among male and more senior students [14]. These findings suggest that unprofessional conduct is common in Pakistani clinical training and may be reinforced within clinical environments, potentially through negative elements of the hidden curriculum.

Medical and dental students share many features of clinical training: common clinical environments, cultural influences, resource constraints, academic governance, and policy frameworks. Although a few studies have included both Bachelor of Medicine, Bachelor of Surgery (MBBS) and Bachelor of Dental Surgery (BDS) learners, none have examined their experiences within the same clinical context [14]. Most research, therefore, treats professions separately, which overlooks opportunities to identify shared drivers of unprofessional behaviour. Moreover, whereas the literature has largely catalogued individual lapses, a behavioural-systems perspective emphasises how organisational and environmental factors shape professional conduct [15]. Therefore, this study aimed to explore final-year medical and dental students' perceptions of shared, system-level drivers of unprofessional conduct during clinical rotations, with the goal of producing practice-oriented recommendations for clinical education. The findings can inform system-aware policies and curricular strategies to strengthen professional development across health professions.

## Materials and methods

This study applied an interpretive description qualitative methodology to explore the understanding and experience of unprofessional behaviours among undergraduate medical and dental students during clinical rotations. Interpretive description was selected because the study aimed to generate practice-oriented insights to inform curriculum and institutional policy. The study was conducted at Bakhtawar Amin Medical and Dental College and Hospital, a private institution in Multan, Pakistan, offering MBBS and BDS programmes. Clinical training is delivered across affiliated teaching hospitals where students interact daily with faculty, house officers, nursing staff, and patients. This naturalistic clinical context provided the conditions under which students commonly witness, interpret, and internalise professional and unprofessional behaviours. A purposive sampling approach was used to recruit participants.

**Figure 1:**
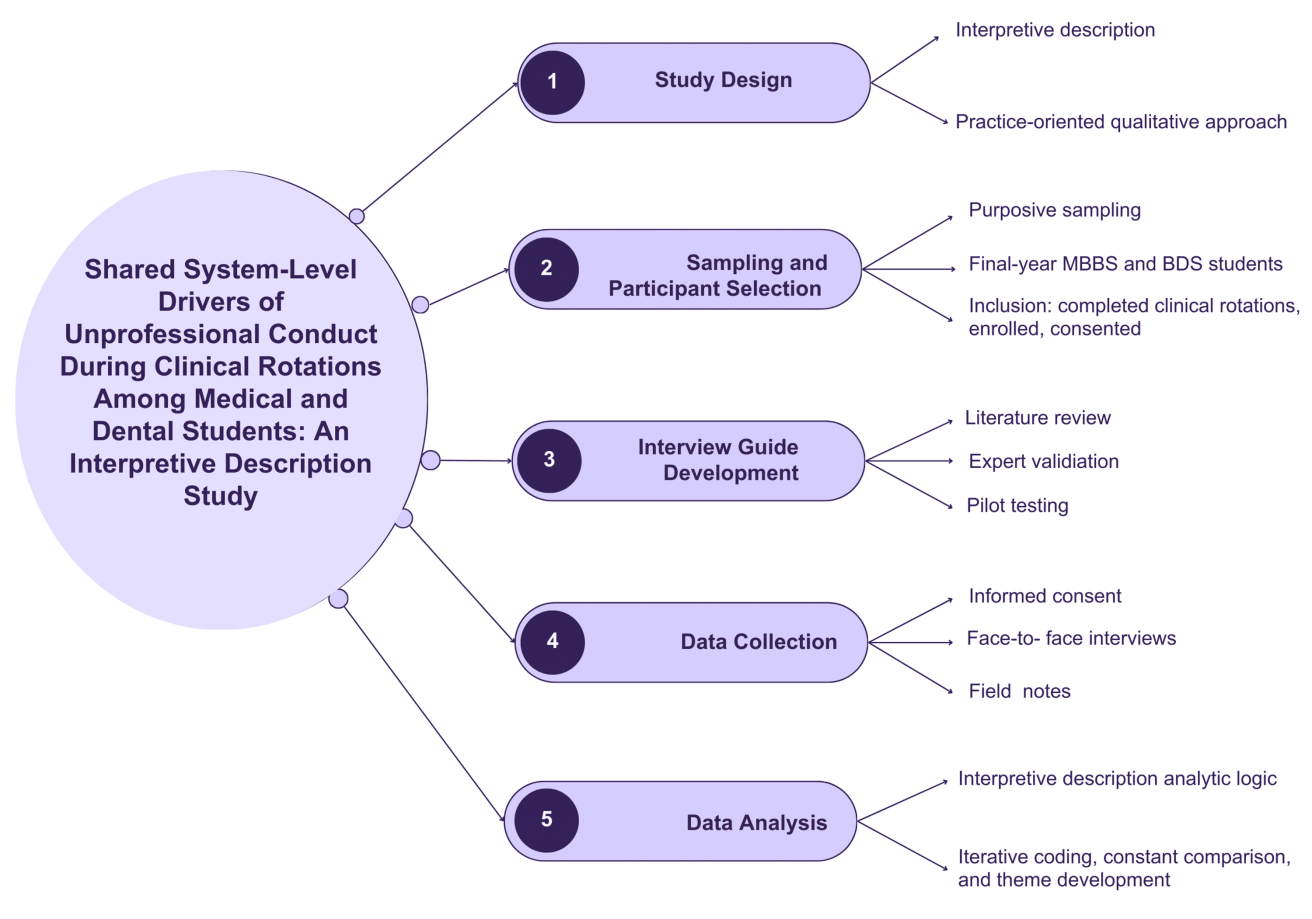
Methodology flowchart Media element by Aida via Canva (https://www.canva.com/) [18] MBBS: Bachelor of Medicine, Bachelor of Surgery; BDS: Bachelor of Dental Surgery

Inclusion criteria were final-year MBBS and BDS students who had completed all clinical rotations at the study institution, were currently enrolled during the study period, and were willing and able to provide written informed consent and permit audio-recording. Exclusion criteria comprised students who had not completed all clinical rotations, those not currently enrolled or on internship, and individuals who declined consent for participation or audio-recording (Figure 1).

The primary interviewer was a faculty member with formal training in qualitative research and experience in health professions education, but was not involved in participant assessment, grading, or promotion decisions. The interviewer's academic role and potential influence on participants were acknowledged and discussed within the research team. Reflexive practices, including the use of neutral prompts, and reassurance of confidentiality were employed to minimise power-related bias during data collection and interpretation. Sampling and analysis occurred concurrently. Between October 2024 and January 2025, 60 final-year medical and dental students were approached and invited to participate. Twenty-four students consented to participate; however, eight were not interviewed due to scheduling constraints related to academic commitments and because interpretive sufficiency had already been achieved. A total of 16 participants, therefore, completed semi-structured interviews. As initial patterns emerged, further recruitment aimed to ensure variability in experiences across clinical departments and gender to enhance interpretive depth. Data collection continued until the interpretive sufficiency was achieved when no new concepts or patterns were emerging during analysis. Practically, this was operationalised as three consecutive interviews producing no novel codes or subthemes and subsequent team review (lead analyst and second coder) confirming that emergent themes captured the meaningful variation in the dataset. Data were collected using semi-structured, in-depth interviews conducted face-to-face in the institution. The interview guide (Appendices) was developed by the research team following a review of the literature on professionalism and unprofessional behaviour [16]. The guide was validated by four field experts, followed by pilot testing for clarity, relevance, and flow. Questions focused on incidents of unprofessional behaviour, the role of the clinical environment, reporting practices, students' responses to informal learning, supervisory role-modelling, participants' meaning-making processes, and reactions to such incidents. Each of the primary questions was followed by specific probes to develop narrative detail, clarify context, and explore differences in experience. The guide encouraged participants to describe specific events, their interpretations of why these occurred, and the institutional conditions shaping these behaviours.

Interviews lasted 28-41 minutes and were audio-recorded with the interviewee's consent. Field notes were recorded after each interview to capture contextual observations and emerging insights. Recordings were transcribed verbatim. Transcripts were anonymised and given study codes. Member checking was limited to factual verification. All digital data were stored on a password-protected device accessible only to the research team. Data were analysed manually using interpretive description analytic logic consistent with interpretive description [17]. The analytic process comprised iterative immersion in transcripts, open coding, constant comparison, analytic memoing, and focused theme development. Two experienced qualitative analysts (MZU and QF) independently coded the transcripts using an inductive approach. Following independent coding of the first three transcripts, the analysts met to compare codes, resolve discrepancies, and refine a shared codebook. All transcripts were subsequently coded using the agreed-upon codebook, with ongoing analytic discussions used to address ambiguities. Initial codes that were developed inductively from the data were then grouped into subthemes, followed by higher-order theme construction through iterative synthesis. Coding was conducted primarily by the lead researcher; a second researcher independently reviewed a subset of transcripts to enhance credibility. Discrepancies were resolved through discussion. An audit trail comprising transcripts, iterative codebooks, and analytic memos was maintained throughout analysis.

The Institutional Research Board of Bakhtawar Amin Medical and Dental College provided ethical approval for the study (approval number: 652/24/COD). Participants provided informed written consent and were assured of confidentiality and voluntary participation. Participants were informed both before and after the interviews about the availability of student counselling services and referral pathways within the institution.

## Results

Table 1 summarises the demographic characteristics of the 16 student participants. The sample comprised an even distribution of male (n=8; 50%) and female (n=8; 50%) participants. Participants were distributed across two age groups; nine (56.3%) were aged 20-25 years, and seven were aged 26-30 years (43.8%). Disciplines were equally represented, with eight (50%) MBBS (medical) students and eight (50%) BDS (dental) students.

**Table 1:** Demographic characteristics of the participants MBBS: Bachelor of Medicine, Bachelor of Surgery; BDS: Bachelor of Dental Surgery

Characteristic	Frequency (n)	Percentage (%)
Gender		
Male	8	50
Female	8	50
Age range (years)		
20-25	9	56.3
26-30	7	43.7
Discipline		
MBBS (medical)	8	50
BDS (dental)	8	50

A total of five themes and 16 subthemes were identified from the analysis: hierarchical culture, hidden curriculum, systemic pressures, erosion of patient-centred care, and student responses to unprofessional conduct (Table 2).

**Table 2:** Medical and dental students' perspectives of unprofessional factors in clinical rotations P: participant; BD: dental; MB: medical; F: female; M: male (1-16): the numeric value is the participant identifier, e.g., (P05BD-F) = participant 05, BDS (dental), female.

Theme	Subtheme	Description	Illustrative quote(s)
Hierarchy and power	Senior doctor authority	Consultants and senior doctors occupy positions of unquestioned authority, within which students are expected to demonstrate compliance.	"Head of Department is the ultimate authority, and most of the seniors consultants are harsh with students, and we are treated and conveyed that we have less knowledge and skill than paramedical staff." (P03MB-M)
	Doctor-nurse hierarchy	Nurses' contributions were often dismissed, raising concerns about their role in patient care.	"Just set the drip and be quiet. You're not a doctor, so don't think." (P08MB-F)
	Social status (class/gender)	Patients' social position affects how they're treated.	"This is what happens when you don’t brush your teeth for months…" (P05BD-F)
Hidden curriculum and norms	Obedience rule	Seniors were treated as unquestionable, and errors were managed privately rather than in an open transparent discussion.	"First and Last rule: The senior is always right, even when wrong… We learnt that reporting a mistake is dangerous." (P01MB-M)
	Compensatory professionalism	Individual ethical responses rather than institutionally supported practices.	"Some clinicians informally support patients through personal acts of generosity, such as paying for medications or procedures when patients could not afford them." (P02MB-F)
	Peer competition	Lack of structured plan and training for learning in clinical rotations.	"We often fight with each other on taking patient turn and sometimes we have to go and search for patients to complete the clinical quota requirements." (P11BD-M)
	Informal mentoring	Some clinicians responded to institutional gaps through informal acts of support, spending extra time teaching outside scheduled hours.	"I've seen consultants during my training who sit with you for an hour and guide your hand through a procedure even after duty hours." (P09BD-M)
Systemic pressures and drivers	Unregulated workload	Multiple overlapping tasks pressure doctors to prioritise speed, undermining empathy.	"He was in a rush expected to see 25-plus patients in a morning, than have lecture and to attend the council meeting…so how we can learn skill or empathy on bedside with such a speed." (P12MB-F)
	Competing commitments	Consultants' competing private practice commitments reduced their availability in teaching clinics.	"I have not seen my HOD in outdoor clinics during my entire clinical rotation… [they] are always busy in private clinics." (P04BD-M)
	Resource scarcity	Limited supervisors and equipment during training.	"In the dental clinic…we often have to start patients with demonstrator or interns as seniors being busy and sometimes have to change the plan due to non-availability of specific materials." (P07BD-M)
Challenges to patient-centred clinical practice	Inadequate patient communication	Patients, particularly those with limited health literacy, are discussed and examined during teaching activities with minimal explanation or active involvement in decision-making.	"Illiterate patients were frequently discussed in front of trainees without clear consent or explanation." (P06MB-F)
	Gender bias	Female students reported being subject to differential expectations and undermining comments that shaped their learning experiences.	"Our… professor asked a female student to act like a man…you have to be strong like men as you selected this specialty." (P08MB-F)
	Clinical objectification	Patients and procedures are seen as educational cases, not people with rights.	"The environment erases patient personhood… You are no longer a person with a history and emotions; you are a pathology." (P01MB-M)
Student impact and coping	Emotional distress	Witnessing or experiencing unprofessional behaviour generated feelings of shame, embarrassment, and moral discomfort about clinical training.	“It made me feel ashamed and uncomfortable. Being shouted publicly in the ward, it also affected my confidence.” [P14MB-F]
	Peer coping strategies	Unable to act, students vent to peers or imitate the behaviour themselves later.	"We respond by gossiping among ourselves… and sometimes by mimicking that behaviour." (P10BD-F)
	Desire for change	Despite fear and silence, students articulated clear suggestions for institutional reform, including safer reporting mechanisms, feedback systems, and faculty development.	"If there were a genuinely confidential system and we saw even one case where speaking up led to change, more students would come forward." (P13BD-F)

One of the major themes that was common in the descriptions of the students was the existence of a strong hierarchical culture in clinical training. Participants described clear, unregulated power where consultants and senior residents were rarely challenged and were perceived as protected by the system. This hierarchy extended beyond doctor-student interactions and influenced relationships with other healthcare staff, particularly nurses. Respondents also reported that underprivileged patients were frequently treated dismissively, sometimes affecting patients' confidence and self-esteem. In various clinical environments, the participants believed that the decision of senior clinicians continued to override the contributions of junior employees or issues regarding patient well-being.

The next theme that was also closely related to this hierarchy was the hidden curriculum that influenced the behaviours and responses of students. Participants described not being encouraged to challenge senior decisions or openly discuss errors; adherence to established norms was commonly perceived as the only route to career advancement. At the same time, students described important contradictions with these norms. Some consultants' financial help to needy patients was perceived by students as informal compensation. Participants also highlighted that a few clinicians provided them additional guidance and mentorship beyond scheduled clinical hours. However, participants also highlighted that the absence of structured training and clear case allocation contributed to frequent peer conflicts over patient management. 

Beyond individual attitudes, participants also highlighted several systemic pressures that enabled unprofessional conduct. Major among these were high patient volumes, academic and research commitments that caused significant time pressure, and limited learning opportunities. Participants also highlighted competing institutional commitments, particularly consultants' involvement in private practice, which reduced their availability for supervision and teaching in training clinics. Limited supervisory capacity, shortages of equipment, and constrained resources further intensified these pressures. 

The effects of hierarchy and systemic pressure frequently manifested as challenges to patient-centred clinical practice across clinical rotations. Several students, regardless of discipline, reported that patients, particularly those with limited health literacy or from lower socioeconomic backgrounds, were discussed or examined during teaching activities without clear explanation or explicit consent, compromising privacy and autonomy. These acts sometimes compromised the privacy and autonomy of patients. Participants described these practices as contributing to a loss of patients' personhood in the clinical setting. In addition, female students reported experiencing gender-based undermining, including being discouraged from pursuing surgical specialties and being perceived as less capable during procedural training.

Participants also mentioned personal and emotional consequences of these experiences. As they reported episodes of public humiliation in clinical settings, particularly when reprimanded openly in wards, which negatively affected their confidence. Coping strategies varied among students; while some withdrew and resolved privately to behave differently in the future, others expressed frustration through unprofessional peer discussions. Despite these experiences, participants across disciplines expressed a desire for change, proposing measures such as anonymous reporting mechanisms, structured faculty and student training, and greater emphasis on communication skills and bedside manner in assessment processes.

## Discussion

This study highlights that unprofessional behaviours in the clinical learning environment arise from certain cultural and structural factors, like rigid hierarchy where seniors are viewed as ultimate decision-makers and questioning them is forbidden, which affects psychological safety and normalises power misuse. These findings align with a 2024 study in the United States that identified multifaceted societal and environmental drivers of unprofessional conduct in clinical settings [19]. The findings suggest that hierarchy operates through two interrelated processes. First, it constrains voice through fear of reputational damage and makes reporting and corrective feedback unlikely. Second, it normalises role-modelling that privileges authority over moral and professional accountability. Therefore, reforms aimed at changing behaviour must address structural features of hierarchy, such as independent review of complaints and leadership accountability. Focusing solely on correcting individual attitudes is insufficient to produce sustained change.

The hidden curriculum emerges as the principal medium through which professional identity is transmitted. Participants described a pattern in which lack of error reporting and conflicts regarding patient cases conveyed tacit lessons that often contradicted formal instruction. The findings are consistent with a study conducted in Iran in 2015 that showed significant cases of lack of error reporting [20]. The study also highlighted individual empathic acts by consultants through helping needy patients and guiding students even beyond duty hours. The findings are endorsed by an editorial commentary advocating bedside role-modelling as central to humane clinical education [21]. The study highlighted that the hidden curriculum adapts in response to system pressures by providing short-term coping strategies; however, if left unchecked, it risks long-term harm to learners' moral development and professional norms. Therefore, curriculum reform should make professionalism explicit through learning outcomes and assessment. Moreover, institutional practices should also include regular facilitated reflection that surfaces and remediates harmful elements of the hidden curriculum.

In addition to norms, students attributed many unprofessional acts to organisational constraints rather than individual intent. Participants considered overwhelming patient volumes, scarce resources, and dual-practice incentives that shift faculty priorities away from teaching. Interestingly, prior work from Canada suggests that intrinsic motivators, particularly connectedness with learners and peers, play a central role in sustaining clinical teachers' engagement, while poorly designed incentive structures may paradoxically undermine motivation [22]. Interpreted through an institutional lens, such behaviours appear less as isolated moral failings and more as functional responses to sustained system pressures. These findings suggest that when faculty are rushed, under-resourced, or structurally incentivised to prioritise private practice, expecting sustained engagement in teaching and role-modelling is unrealistic. Therefore, institutional audits of workload, protected teaching time, realistic student quotas, and explicit job descriptions that safeguard teaching duties should be prioritised.

The combined effect of hierarchy, hidden norms, and system stressors produced profound distress among students. Witnessing or experiencing gender bias or patient mistreatment left learners discouraged. This mirrors a 2021 study reporting that mistreated trainees suffer guilt and eroded confidence [23]. These incidents emerged when educational demands and systemic pressures converged, reinforcing the normalisation of compromised ethical standards. To counter these effects and protect the dignity of patients and students, we recommend concrete educational measures such as requiring explicit informed consent for bedside teaching, enforcing clear expectations for privacy during clinical encounters, and implementing assessment frameworks that formally evaluate respectful patient communication.

These insights highlight the need to protect student well-being as a core institutional goal. Participants described significant emotional distress and maladaptive coping responses, including withdrawal and, at times, the replication of unprofessional behaviours. This pattern is consistent as a study conducted in Pakistan in 2020 also revealed emotional distress in undergraduate medical students due to public insult [24]. Despite these challenges, students expressed a clear desire for change, specifically requesting improvements in feedback systems, evaluation processes, and structured clinical training. Previous literature also endorsed these findings [25]. The findings suggest that health professions programmes must cultivate psychological safety so learners can raise concerns without fear of retaliation or harm to their emotional well-being. Institutions should therefore facilitate confidential reporting, mentorship programmes pairing junior students with positive role models, and formalised debriefing after ethically compromised encounters.

This study's inclusion of final-year medical and dental students offers a cross-professional perspective that enhances the relevance of findings for integrated clinical education reform. Emphasising system-level factors provided contextually grounded, policy-relevant insights for low- and middle-income settings. The use of an interpretive description approach aligned with our practice-oriented aims; purposive sampling captured diverse clinical exposures; and pilot-tested, expert-validated interview guides further strengthened the study. Finally, the study produced actionable recommendations including protected teaching time, confidential reporting mechanisms, and faculty development that increase its practical utility for institutional reform.

Implications for clinical education and practice

The findings have direct implications for clinical education and institutional policy. Institutions should protect and audit dedicated teaching time and set realistic clinical quotas to reduce time-pressured supervision that fosters shortcuts and disrespectful practice. Confidential reporting mechanisms, regular facilitated reflection sessions, and structured mentorship should be established to surface hidden curriculum harms and support affected learners. Finally, routine clinical evaluations should explicitly assess respectful communication, informed consent for bedside teaching, and role-modelling by supervisors so that incentives and assessment practices align with the professional behaviours expected of staff and trainees.

Limitations

The study was conducted at a single private institution, which limits empirical transferability to other types of clinical settings. Public-sector clinical settings in Pakistan commonly differ in supervisory structures, patient volumes, resource availability, and clinician incentive arrangements; these contextual differences could alter how system-level drivers of unprofessional conduct manifest. Data were collected only from students; faculty, nurses, and patients were not interviewed, constraining the triangulation of perspectives on institutional drivers and behaviours. Reliance on retrospective interview accounts introduces recall and social desirability biases; organisational claims were participant-reported rather than independently measured. Rather than claiming generalisability, we emphasise analytic transferability and have provided detailed contextual information so that readers can judge whether the findings apply to their setting. Accordingly, the system-level drivers described should be interpreted as student-perceived institutional influences derived from experiential accounts rather than as objective or independently verified measures of organisational practice.

Future multi-site, mixed-method studies that incorporate longitudinal follow-up are needed to assess transferability and to examine how sustained exposure to clinical culture influences professional identity formation over time.

## Conclusions

Unprofessional behaviours observed during clinical rotations are not isolated lapses but the product of unchecked and unregulated power, a pervasive hidden curriculum that negatively shapes professional identity, and system-level pressures that together erode student well-being, professional development, and patient dignity. Addressing these problems requires coordinated institutional action to protect and value teaching time, establish confidential reporting and remediation pathways, invest in sustained faculty development focused on respectful supervision and role-modelling, and incorporate explicit curricular strategies that surface and counteract the unprofessional aspects of hidden curriculum. Implementing these measures can restore psychological safety for learners, safeguard patient dignity during clinical teaching, and better align everyday practice with the stated values of clinical education. These recommendations should be understood as participant-informed and analytically derived rather than as evidence-based or evaluated interventions.

## Appendices

**Table 3:** Semi-structured interview guide

S. no.	Interview question
1	Can you describe an instance during your clinical rotations when you observed or personally experienced behavior you considered unprofessional? Please explain what happened and who was involved.
2	In your view, what aspects of the clinical training environment contribute to or allow unprofessional behavior to occur?
3	How have supervisors or mentors (such as attending physicians, senior dentists, or residents) modeled professional or unprofessional behavior during your training? Can you share examples of how their behavior affected you or your peers?
4	What implicit messages have you learned from the clinical environment about what is acceptable or expected behavior?
5	When you reflect on unprofessional incidents you have witnessed, how do you interpret why they occurred? What factors do you think caused or enabled such behavior?
6	How have you or others typically responded when witnessing unprofessional behavior? Have you ever reported it, confronted it, or chosen not to act? What factors influenced your decision?
7	What is your understanding of the formal processes for reporting unprofessional conduct in your program?
8	Based on your experiences, what system-level or institutional changes do you think could promote professionalism and reduce unprofessional conduct during clinical training?
